# Real‐World Use of Avacopan in Antineutrophil Cytoplasmic Antibody–Associated Vasculitis: Early and Long‐Term Clinical, Renal, and Patient‐Reported Outcomes in an Italian Rheumatology–Nephrology Network

**DOI:** 10.1002/acr2.90126

**Published:** 2026-10-11

**Authors:** Luca Quartuccio, Elena Treppo, Miriam Isola, Maria De Martino, Chiara Marvisi, Luca Iorio, Alessandra Milanesi, Luca Moroni, Edoardo Conticini, Stefania Affatato, Maria Letizia Urban, Enrico Tombetti, Fabio Barone, Pasquale Esposito, Florenzo Iannone, Valentina Binda, Giuseppe Castellano, Corrado Murtas, Lida Tartaglione, Francesco Delle Monache, Amelia Rigon, Maria Chiara Panini, Marco Allinovi, Augusto Vaglio, Bruno Frediani, Paolo Delvino, Sara Monti, Roberto Padoan, Carlomaurizio Montecucco, Lorenzo Dagna, Giacomo Emmi, Federico Alberici, Carlo Salvarani, the Italian Society of Rheumatology (Vasculitis and Polymyalgia Rheumatica Study Group)

**Affiliations:** ^1^ Division of Rheumatology, Department of Medicine University of Udine Udine Italy; ^2^ Department of Medical Area, Institute of Statistics University of Udine Udine Italy; ^3^ Azienda USL‐IRCCS di Reggio Emilia and University of Modena and Reggio Emilia Reggio Emilia Italy; ^4^ Rheumatology Unit, Department of Medicine DIMED University of Padua Padova Italy; ^5^ Division of Rheumatology, Department of Internal Medicine and Therapeutics, IRCCS Policlinico San Matteo University of Pavia Pavia Italy; ^6^ Unit of Immunology, Rheumatology, Allergy and Rare Diseases (UnIRAR) IRCCS San Raffaele Scientific Institute Milano Italy; ^7^ Rheumatology Unit, Department of Medicine, Surgery and Neurosciences University of Siena Siena Italy; ^8^ Nephrology Unit Università di Brescia, ASST Spedali Civili Brescia Italy; ^9^ Department of Medical, Surgery and Health Sciences University of Trieste; ^10^ Clinical Medicine and Rheumatology Unit Cattinara University Hospital Trieste Italy; ^11^ Internal Medicine and Rheumatology, Department of Biomedical and Clinical Sciences Sacco and Fatebenefratelli Hospitals Milan Italy; ^12^ Department of Internal Medicine and Medical Specialties (DIMI) University of Genoa Genova Italy; ^13^ Unit of Nephrology, Dialysis and Transplantation, IRCCS Azienda Ospedaliera Metropolitana Policlinico San Martino Genova Italy; ^14^ DiMePReJ ‐ Rheumatology Unit University of Bari Bari Italy; ^15^ Department of Nephrology, Dialysis and Kidney Transplantation Fondazione IRCCS Ca’ Granda Ospedale Maggiore Policlinico Milan Italy; ^16^ UOC Nephrology and Dialysis ASL Viterbo Viterbo Italy; ^17^ Unit of Nephrology, Dialysis and Transplantation AOU Umberto I Roma Italy; ^18^ Internal Medicine Unit, Medical Department G. Mazzini Hospital Teramo Italy; ^19^ Clinical and Research Section of Rheumatology and Clinical Immunology Fondazione Policlinico Universitario Campus Bio‐Medico Rome Italy; ^20^ Department of Nephrology, Dialysis and Transplantation Unit Careggi University Hospital Florence Italy; ^21^ Nephrology Unit Meyer Children's Hospital IRCCS Florence Italy; ^22^ Department of Biomedical, Experimental and Clinical Science University of Florence Florence Italy; ^23^ School of Medicine and Surgery, University of Milano‐Bicocca Milan Italy; ^24^ Rheumatology Unit Fondazione IRCCS San Gerardo dei Tintori Monza Italy; ^25^ IRCCS Istituto Auxologico Italiano, Unit of Immunology, Allergology and Rheumatology Milan Italy; ^26^ Department of Clinical Sciences and Community Health University of Milan Milan Italy; ^27^ Centre for Inflammatory Diseases Monash University Department of Medicine, Monash Medical Centre Melbourne VIC Australia

## Abstract

**Objective:**

Complement activation, particularly through the C5a–C5a receptor axis, plays a pivotal role in the pathogenesis of antineutrophil cytoplasmic antibody–associated vasculitis (AAV) and may represent an important therapeutic target. Avacopan, an oral C5a receptor antagonist, was the first therapy targeting this pathway to enter in clinical practice. Prospective real‐world data on its long‐term effectiveness, safety, and impact on quality of life (QoL) remain limited. In the context of an evolving therapeutic and regulatory landscape, such data provide valuable evidence on the clinical effects of C5a receptor inhibition and may inform the development and evaluation of future complement‐targeted therapies. The objective of this study was to evaluate the real‐world effectiveness, safety, kidney outcomes, and patient‐reported outcomes of avacopan in an Italian cohort of patients with granulomatosis with polyangiitis (GPA) or microscopic polyangiitis (MPA).

**Methods:**

This prospective, multicenter, observational study included patients with newly diagnosed or relapsing GPA or MPA who were started on avacopan therapy across 18 Italian centers. Avacopan was administered in combination with standard immunosuppressive therapy, with or without glucocorticoids (GCs). Clinical remission (Birmingham Vasculitis Activity Score = 0), GC‐free remission, kidney parameters, laboratory outcomes, adverse events, and patient‐reported outcomes assessed by the AAV Patient‐Reported Outcome questionnaire were evaluated up to 52 weeks and beyond. Estimated glomerular filtration rate (eGFR) was calculated using the European Kidney Function Consortium equation.

**Results:**

Forty‐six patients were included (63% women; mean age of 59 years); 61% had relapsing disease, and 70% had kidney involvement at baseline. Clinical remission was achieved by 78%, 88%, and 97% of patients at weeks 12, 26, and 52, respectively. GC‐free remission occurred in 20% at week 12 and 55% at week 52. Mean GC dose decreased from 23 mg/day at baseline to 5 mg/day by week 12 (*P* < 0.001). Kidney function improved over time, with median eGFR increasing from 30.5 mL/min/1.73 m^2^ at baseline to 53.5 mL/min/1.73 m^2^ at week 52 (*P* = 0.002). Significant improvements in QoL were observed as early as week 12 and were sustained until week 52, particularly in the physical function domain. Adverse events were mild to moderate; infections were the most frequent events (40%). Disease relapses were uncommon and occurred exclusively in patients treated for relapsing disease.

**Conclusion:**

In this real‐world Italian cohort, patients treated with avacopan achieved high remission rates, substantial GC reduction, improvement in kidney function, and sustained QoL benefits. These findings provide prospective real‐world evidence on the clinical outcomes associated with C5a receptor inhibition in routine clinical practice while acknowledging the observational design of the study.

## INTRODUCTION

Antineutrophil cytoplasmic antibody (ANCA)‐associated vasculitis (AAV), including granulomatosis with polyangiitis (GPA), microscopic polyangiitis (MPA), and eosinophilic granulomatosis with polyangiitis (EGPA), are rare, potentially life‐threatening autoimmune diseases characterized by necrotizing inflammation of small‐ to medium‐sized blood vessels. Despite advances in treatment with immunosuppressive agents and glucocorticoids (GCs), long‐term GC toxicity remains a major concern.

**SIGNIFICANCE & INNOVATIONS**

- Patients treated with avacopan achieved high and sustained remission rates in this real‐world cohort, with nearly all patients reaching clinical remission at one year and more than half of patients reaching glucocorticoid‐free remission.
- Renal function and patient‐reported quality of life improved early after treatment initiation and remained stable during follow‐up.
- Avacopan was generally well tolerated in this predominantly relapsing cohort, supporting its feasibility in routine rheumatology–nephrology practice.

Avacopan, an oral selective C5a receptor antagonist, was developed as a GC‐sparing therapy for GPA and MPA. Its efficacy and safety were initially supported by the randomized‐controlled ADVOCATE trial, leading to regulatory approval in several countries, including the United States and Europe. However, the original publication of the ADVOCATE trial was subsequently retracted by the *New England Journal of Medicine* following concerns regarding the integrity of the underlying clinical data.^1^ Therefore, evidence from independent prospective real‐world studies is particularly important to assess the effectiveness and safety of complement inhibition in routine clinical practice. Although randomized clinical trials provide the basis for regulatory approval, prospective real‐world studies remain essential to describe clinical outcomes, safety, and patient‐reported quality of life (QoL) in unselected patient populations. This study aimed to evaluate the clinical outcomes, safety, and patient‐reported outcomes of avacopan in a prospective real‐world Italian cohort of patients with GPA or MPA, including both those treated within the Early Access Program and those who initiated therapy following regulatory approval.

## MATERIALS AND METHODS

### Study design and population

This was a prospective, multicenter, observational study conducted across 18 centers in Italy. Patients with either newly diagnosed or relapsing GPA or MPA who were initiated on avacopan therapy between May 2022 and October 2024 were enrolled. The baseline was defined as the time of avacopan initiation.

### Treatment and follow‐up

All patients received avacopan in combination with standard immunosuppressive agents, namely rituximab (RTX) and/or cyclophosphamide (CYC), as per physician judgment. Concomitant GC therapy was allowed, and doses were recorded. Clinical assessments were performed at baseline; weeks 4, 8, 12, 16, and 26; and then every 12 weeks until the last available follow‐up. Disease activity and laboratory parameters (including blood cell counts, liver and kidney function tests, and C‐reactive protein [CRP]) levels were collected at each time point. Participants completed the validated Italian version of the AAV Patient‐Reported Outcome (AAV‐PRO_ita) questionnaire every 12 weeks.^2^ Vasculitis Damage Index (VDI) was collected at baseline and at 52 weeks.

Clinical remission was defined as a Birmingham Vasculitis Activity Score (BVAS) of 0, whereas GC‐free clinical remission was defined as a BVAS of 0 with no GC use in the preceding four weeks. Relapse was defined as the reappearance of any symptoms and signs related to AAV (BVAS > 0) requiring the increase in GC dose and/or the intensification of immunosuppressive treatment. All adverse events (AEs), as well as both permanent and temporary treatment discontinuations, were recorded. Estimated glomerular filtration rate (eGFR) was calculated using the European Kidney Function Consortium equation. The study was approved by the ethics committee of the coordinating site (CEUR‐2024‐Os‐182).

### Primary and secondary endpoints

The aim of the study was to evaluate the real‐world efficacy, safety, and impact on QoL of avacopan in an Italian cohort of patients treated at expert rheumatology and nephrology centers. The primary endpoint was the proportion of patients achieving GC‐free clinical remission at weeks 12, 26, and 52.

Secondary endpoints included the proportion of patients achieving clinical remission at weeks 12, 26, and 52. Safety was evaluated by assessing AEs in a heterogeneous patient population treated outside the setting of controlled clinical trials. Renal outcomes were analyzed by assessing early changes in kidney function parameters, including serum creatinine, proteinuria, and microhematuria. In addition, changes in QoL were measured using the AAV‐PRO_ita questionnaire. Domain scores were calculated by summing the items within each of the three domains—specific/systemic symptoms (SSS), physical function (PF), and social and emotional impact (SEI)—and linearly transforming the totals to a 0–100 scale, with higher scores indicating greater disease impact and worse health‐related QoL.

### Statistical analysis and artificial intelligence statement

Descriptive statistics were used to summarize baseline characteristics and treatment outcomes. Changes in continuous variables (eg, BVAS, kidney function, and AAV‐PRO_ita domain scores) were assessed using paired *t*‐tests or Wilcoxon signed‐rank tests, as appropriate based on data distribution. Data distribution was assessed using the Shapiro–Wilk test. Categorical variables (eg, remission rates, AEs) were compared using chi‐square tests or Fisher's exact tests. A *P* value <0.05 was considered significant. Artificial intelligence tools were used to generate some images included in the manuscript.

### Data availability statement

The data supporting the findings of this study are available from the corresponding author upon reasonable request.

## RESULTS

### Baseline characteristics

A total of 46 patients were included (29 with GPA and 17 with MPA); their mean age was 59 years (SD 14). Sixty‐three percent of patients were women, and 93% were Caucasian. ANCA positivity and biopsy‐proven AAV diagnosis were observed in 98% and 72% of patients, respectively. Proteinase 3–ANCA were detected in 29 of 46 patients and myeloperoxidase‐ANCA in 16 of 46. In the patient who was ANCA negative, the diagnosis was confirmed by skin biopsy. Eighteen of 46 (39%) patients had newly diagnosed disease, whereas the remaining 28 of 46 (61%) were experiencing a relapse (mean disease duration: 9 years [SD 6.5]), of whom 79% (22 of 28) were GC dependent at baseline.

Avacopan was administered in combination with RTX in 38 patients, with CYC in 1 patient, or with both RTX and one or two intravenous pulses of CYC (mean cumulative dose: 1,613 mg [SD 1,497]) in 7 patients. Three patients underwent plasmapheresis. At baseline, the mean dose of GCs was 23 mg/day (SD 19), with one newly diagnosed patient initiating avacopan without GCs. Baseline BVAS and VDI scores were 10 (SD 8) and 3 (SD 3), respectively. Systemic symptoms (72%) and kidney involvement (70%) were the main disease manifestations, as shown in Figure 1. Severe manifestations, such as rapidly progressive glomerulonephritis and hemorrhagic alveolitis, were present in 43% and 13% of patients, respectively. Among patients with kidney involvement (n = 32), the median eGFR at baseline was 30.5 mL/min/1.73 m^2^ (interquartile range [IQR] 18.0–42.4). There were 14 of 32 patients who had an eGFR <30 mL/min/1.73 m^2^, including 2 patients with an eGFR <15 mL/min/1.73 m^2^. One of these patients was receiving maintenance dialysis at the time of avacopan initiation.

**Figure 1:**
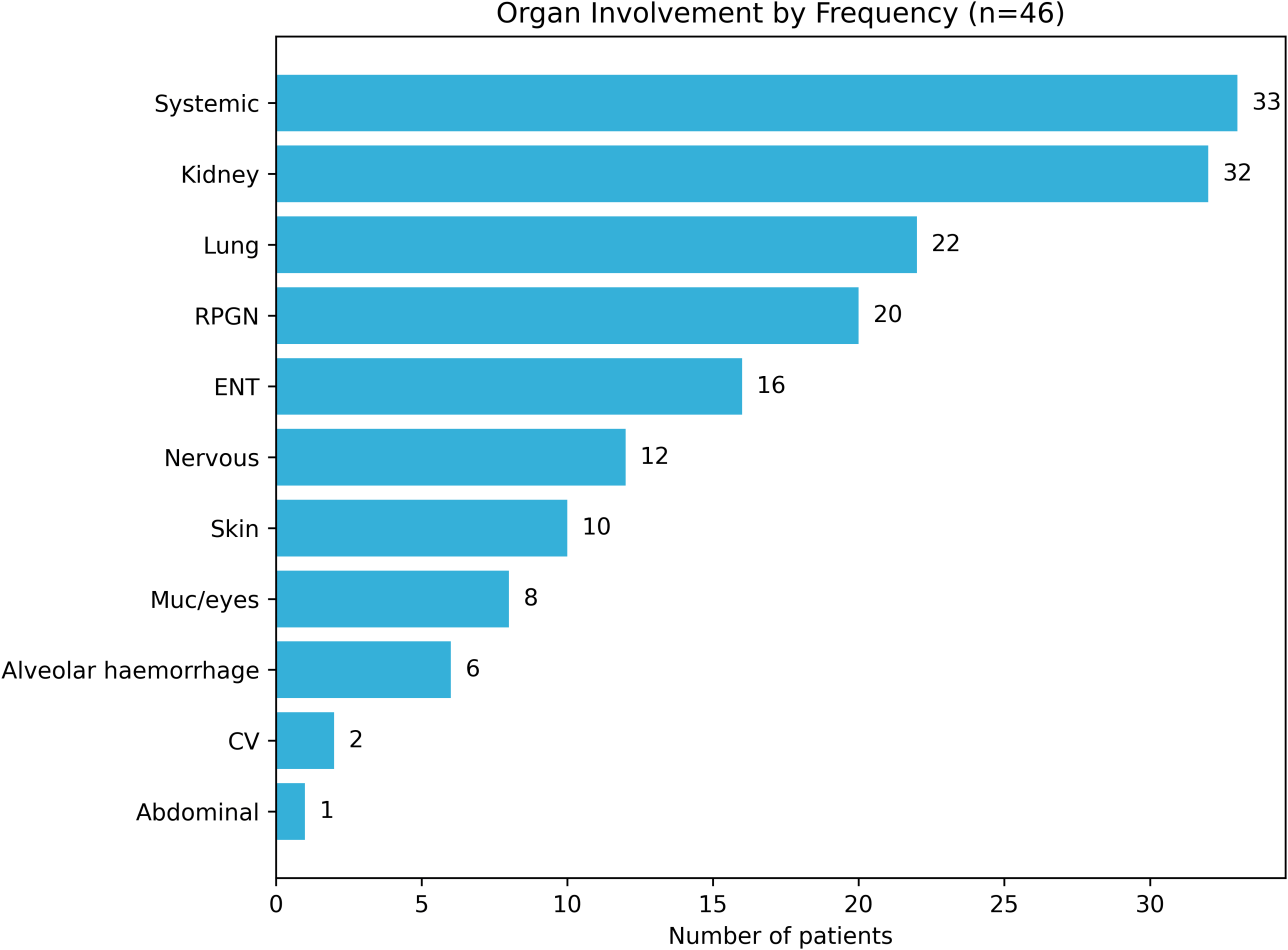
Disease involvement at baseline. CV, cardiovascular; ENT, ear‐nose‐throat; Muc/eyes, mucous and eyes involvement; RPGN, rapidly progressive glomerulonephritis.

### Early outcomes (week 12)

At week 12, 9 of 46 patients (20%) were in GC‐free clinical remission, whereas 36 patients (78%) achieved clinical remission. The mean GC dose declined from 23 mg/day to 5 mg/day (SD 6; *P* < 0.001). Kidney function significantly improved among the 32 patients with kidney impairment at baseline. Specifically, serum creatinine levels decreased from 2.25 mg/dL (SD 1.13) to 1.76 mg/dL (SD 0.83; *P* = 0.009), and proteinuria levels decreased from 1,640 mg/24 hours (SD 1,703) to 902 mg/24 hours (SD 1,273; *P* = 0.004). Hematuria was observed in 84% (27 of 32) of patients at baseline and in 38% (12 of 32) at week 12 (*P* < 0.001).

An early improvement in QoL was already observed by week 12 (Figure 2). AAV‐PRO_ita scores decreased across all domains (SSS, PF, and SEI) compared to baseline. Median scores of SSS, PF, and SEI significantly decreased from 36.4 (IQR 20.5–54.5), 45.0 (IQR 25.0–70.0), and 50.0 (IQR 25.0–69.2) at baseline to 21.6 (IQR 11.4–33.5; *P* = 0.003), 27.5 (IQR 20.0–42.5; *P* = 0.003), and 32.7 (IQR 14.9–60.1; *P* = 0.025) at week 12, respectively.

**Figure 2:**
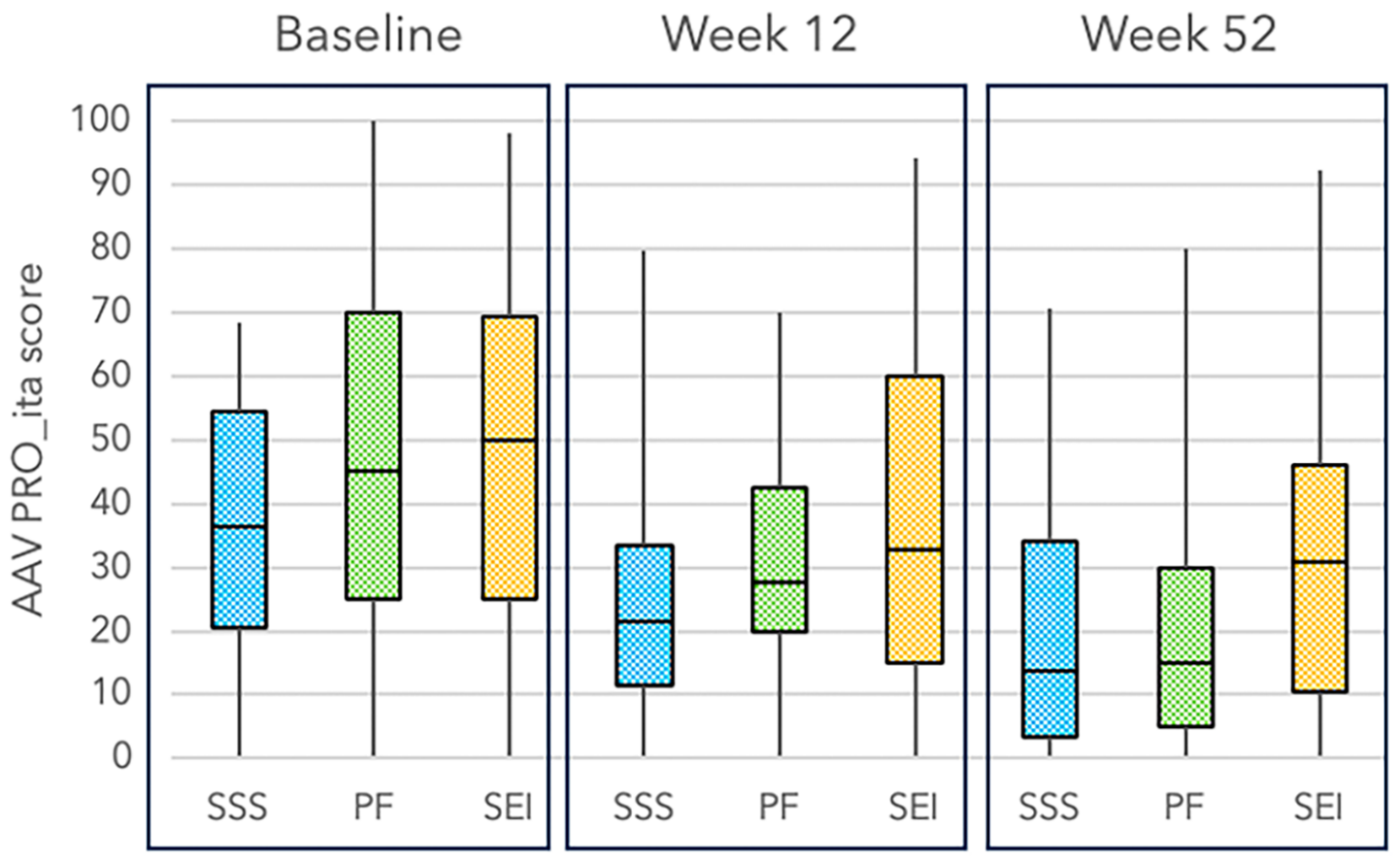
Median score changes (0–100) of the AAV‐PRO_ita questionnaire across the three domains (SSS, PF, SEI) at baseline, week 12, and week 52. AAV‐PRO, Antineutrophil cytoplasmic antibody–Associated Vasculitis Patient‐Reported Outcome; PF, physical function; SEI, social and emotional impact; SSS, systemic/specific symptoms. *P < 0.05 and **P < 0.01 vs baseline.

### Long‐term follow‐up

The mean duration of avacopan therapy was 61 weeks (SD 34), with 39% of patients continuing treatment beyond one year (Figure 3). GC‐free clinical remission was observed in 37% (15 of 41) of patients at week 26 and in 53% (17 of 32) at week 52. Clinical remission was achieved in 88% (36 of 41) of patients at week 26 and in 97% (31 of 32) at week 52. At week 52, GC‐free remission was achieved in 75% of newly diagnosed patients compared with 40% of relapsing patients (9 of 12 vs 8 of 20; odds ratio 4.5, 95% confidence interval 0.9–22; *P* = 0.07). The VDI did not change over time, remaining stable from baseline to 12 months (2 [IQR 0–3] vs 2 [IQR 1–3]; Wilcoxon signed‐rank test *P* = 0.25). At week 104, eight patients were still under active follow‐up and continued avacopan therapy, whereas five others had discontinued treatment between weeks 52 and 91 of follow‐up due to sustained remission and physicians’ decision and remained in clinical remission off therapy. Overall, 76% (35 of 46) of patients received maintenance therapy with RTX during follow‐up, most commonly 500 mg every six months (74%; 26 of 35), followed by 500 or 1,000 mg every six months (14%; 5 of 35), 1,000 mg every six months (6%; 2 of 35), and 1,000 mg administered two weeks apart every six months (6%; 2 of 35).

**Figure 3:**
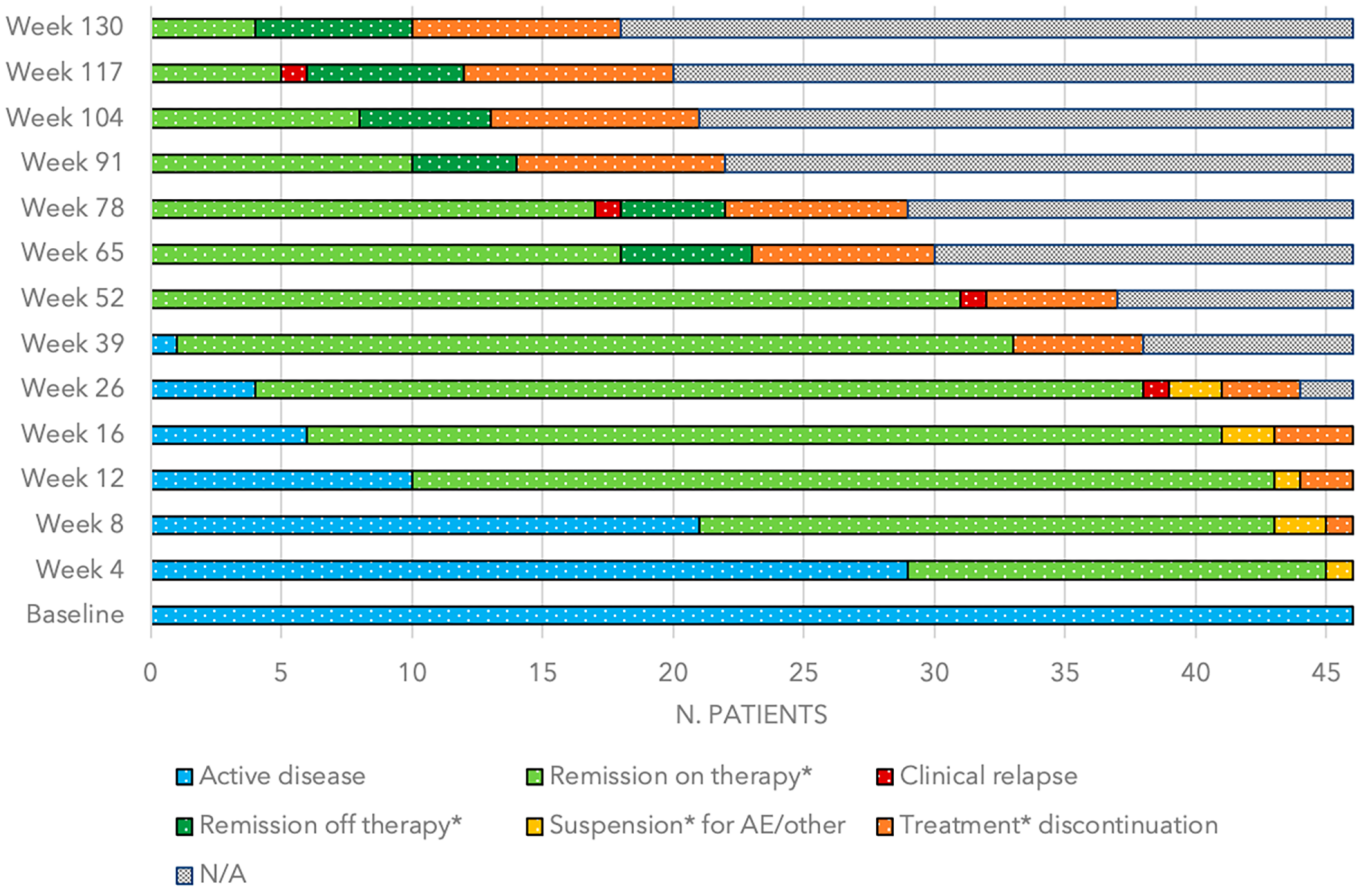
Treatment outcomes with avacopan. *Referred to avacopan therapy. AE, adverse event; N/A, not applicable.

The improvement in QoL was still evident at week 52 (Figure 2). At week 52, median scores of SSS, PF, and SEI were 13.6 (IQR 3.4–34.1), 15.0 (IQR 5.0–30.0), and 30.8 (IQR 10.6–46.2), respectively. These scores showed a statistically significant improvement compared with baseline (*P* = 0.002, *P* = 0.003, and *P* = 0.002), whereas, compared with week 12, the scores remained stable for SSS and SEI (*P* = 0.121 and *P* = 0.073, respectively), with a further significant improvement observed in the PF domain (*P* = 0.040).

### Relapses and safety profile

Among 46 patients, four relapses were recorded in three individuals at weeks 26, 52, 78, and 117, respectively. All three had initiated avacopan therapy for relapsing disease. A 32‐year‐old man and a 65‐year‐old woman experienced relapses of subglottic stenosis. The former had two recurrences during follow‐up, managed with GCs and local therapy. A 78‐year‐old woman developed a recurrence of alveolar hemorrhage and kidney function impairment requiring RTX administration; unfortunately, she developed a fungal infection and died at week 60.

Overall, nine patients (20%) discontinued treatment due to relapse (two patients), infections (four patients), initiation of dialysis (one patient), comorbidities (one patient), or patient decision (one patient; Table 1). The latter three, along with another patient who discontinued due to a viral infection, stopped avacopan early (ie, within 16 weeks). The median time from treatment initiation to discontinuation was 26 weeks (IQR 12–52). Infections causing avacopan discontinuation included airway infections (two patients), urinary tract infection (one patient), and acute cholecystitis (one patient). A 72‐year‐old woman who discontinued avacopan at week 39 for AEs (initially a respiratory infection, followed by gastrointestinal intolerance [ie, diarrhea and abdominal pain]) died from bacterial pneumonia 13 months later. Overall, eight hospitalizations occurred in six patients during avacopan treatment. The causes included active disease (four patients), infections (three patients), and conditions unrelated to AAV (one patient).

**Table 1:** Adverse events recorded during avacopan treatment*

Category	Cause	Number of patients, N
Permanent discontinuation	Overall infections	4
	Airway infections	2
	Urinary tract infection	1
	Acute cholecystitis	1
	Relapse	2
	Initiation of dialysis	1
	Comorbidities	1
	Patient decision	1
Temporary discontinuation	Airway infections	3
	Temporary unavailability of medication	2
	Diarrhea	1
	Urinary tract infection	1
	Scheduled surgery	1
Other adverse events (no discontinuation)	Grade 1 liver enzyme elevation (CTCAE)	2
	Grade 1 leukopenia (CTCAE)	1

* CTCAE, Common Terminology Criteria for Adverse Events.

Eight temporary discontinuations of avacopan were recorded in seven patients (Table 1). The median time of interruption was three weeks (IQR 3–4). The main cause was airway infection (three patients), followed by temporary unavailability of the medication (two patients), diarrhea (one patient), urinary tract infection (one patient), and scheduled surgery (one patient). Other AEs recorded that did not require treatment discontinuation included mild elevations in liver function tests (in one instance, the avacopan dose was temporarily reduced to 10 mg twice daily; in another, no intervention was needed) and grade 1 leukopenia (one patient).

### Laboratory parameters and kidney outcomes

At the initiation of avacopan therapy, almost all patients were ANCA positive, whereas at week 52, slightly more than one‐third of patients remained ANCA positive (Figure 4A). In parallel, systemic inflammatory activity progressively decreased during follow‐up. Mean CRP levels declined early after treatment initiation, showing a downward trend at week 4 (*P* = 0.065) and week 12 (*P* = 0.069), reaching a statistically significant reduction at week 26 (*P* = 0.012; Figure 4B).

**Figure 4:**
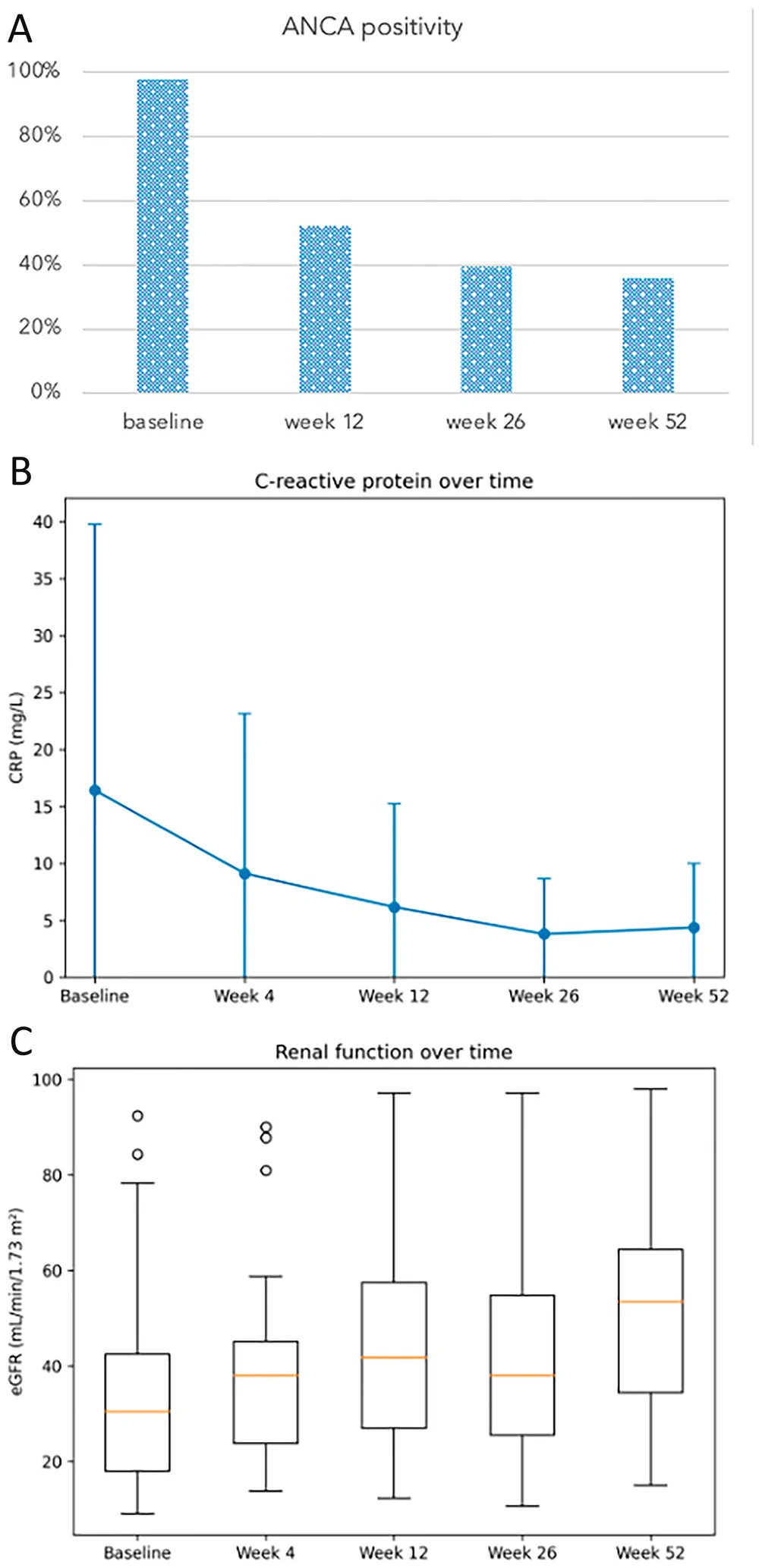
Laboratory and kidney outcomes over time. (A) Proportion of patients who were ANCA positive at baseline and at weeks 12, 26, and 52 after initiation of avacopan therapy. (B) Mean CRP levels at baseline and at weeks 4, 12, 26, and 52; data are shown as mean ± SD. (C) Median eGFR at baseline and during follow‐up; data are shown as median and interquartile range. ANCA, antineutrophil cytoplasmic antibody; CRP, C‐reactive protein; eGFR, estimated glomerular filtration rate.

Among patients with kidney involvement, kidney function improved over time. The greatest improvement occurred during the first 12 weeks of treatment, followed by maintenance of this improvement throughout subsequent follow‐up, with the highest median value observed at week 52 (Figure 4C). Median eGFR increased from 30.5 mL/min/1.73 m^2^ (IQR 18.0–42.4) at baseline to 38.0 (IQR 23.8–45.2) at week 4 (*P* = 0.080), 41.8 (IQR 27.0–57.5) at week 12 (*P* = 0.0009), 38.0 (IQR 25.6–54.8) at week 26 (*P* = 0.015), and 53.5 (IQR 34.4–64.5) at week 52 (*P* = 0.002). Overall, one patient progressed to dialysis‐dependent kidney failure during follow‐up, whereas one patient was already receiving maintenance dialysis at baseline and remained dialysis‐dependent throughout follow‐up.

## DISCUSSION

This prospective, multicenter, real‐world study provides insights into the effectiveness, safety, and QoL impact of avacopan in Italian patients with GPA and MPA. Unlike the protocol‐driven ADVOCATE trial, our study reflects routine clinical practice, and three‐quarters of our patients received maintenance treatment with RTX during follow‐up. This observation supports the feasibility and safety of combining avacopan with repeated RTX maintenance after induction.^3^ Moreover, in our cohort, the majority of patients were experiencing a relapse (61%), a long‐standing disease duration, and a high rate of GC dependence at baseline, highlighting the greater clinical complexity of our cohort compared with that of the pivotal trial.

GC exposure remained higher throughout follow‐up than that reported in ADVOCATE. This difference likely reflects the observational nature of our study, in which GC tapering was left to the treating physician's discretion rather than being protocol mandated, together with the greater disease complexity of our population. Most patients had relapsing disease, long‐standing disease, and a high prevalence of GC dependence at baseline, all of which may have contributed to a more cautious tapering strategy. Nevertheless, GC exposure declined substantially during follow‐up, with the mean dose decreasing from 23 mg/day at baseline to 5 mg/day by week 12, and more than half of evaluable patients achieving GC‐free remission at week 52. These findings highlight both the GC‐sparing potential of C5a receptor inhibition and the challenges of implementing GC withdrawal in routine clinical practice. At week 52, GC‐free remission was achieved in 75% of newly diagnosed patients compared with 40% of relapsing patients (*P* = 0.07). Although this difference did not reach statistical significance, possibly due to the limited sample size, it may suggest that patients treated at disease onset could derive greater benefit from early C5a receptor inhibition in terms of sustained GC‐free remission. Clinical remission rates at weeks 26 and 52 were comparable to those reported in the largest published real‐world cohort by Zonozi et al and are consistent with findings from other independent observational studies.^4^ Although comparisons with the ADVOCATE trial should be interpreted with caution following the retraction of its original publication and the subsequent regulatory investigations, the consistency across independent real‐world cohorts supports the reproducibility of the clinical outcomes associated with C5a receptor inhibition in routine practice.

Furthermore, it should be remembered that GC‐sparing strategy may also influence patients’ perception of QoL.^2^ A previous study demonstrated that improvements in AAV‐PRO_ita questionnaire scores were greater in patients who were GC free, particularly in the PF domain (*P* < 0.001).^2^ Intriguingly, it was specifically the PF domain that showed a progressive improvement over time in our cohort. The progressive improvement in PF may reflect both disease control and reduced GC exposure.

Regarding kidney outcomes, our findings are consistent with previously reported evidence on complement inhibition in AAV.^1^, ^5^ Among patients with baseline kidney involvement (70%), improvements in serum creatinine, proteinuria, and hematuria were evident by week 12 and sustained throughout follow‐up. In addition, kidney function showed a progressive improvement over time, as reflected by an increase in eGFR, with an early upward trend followed by a significant improvement by week 12 and subsequent stabilization up to 52 weeks (Figure 4C). These findings are consistent with previous evidence supporting the early renal benefits of C5a receptor inhibition and suggest sustained improvement in kidney inflammation and functional recovery for up to 12 months.^5^

Despite the suggested efficacy of avacopan in life‐threatening manifestations,^6^ relapses in our cohort mainly involved recurrence of subglottic stenosis and renal‐pulmonary syndrome, highlighting the persistent therapeutic challenges associated with these manifestations. Relapses were infrequent and confined to patients treated for relapsing disease, suggesting that achieving sustained remission may be more difficult in this subgroup, particularly in those with airway or pulmonary involvement. Notably, 39% of patients continued avacopan beyond one year, and some maintained remission even after treatment discontinuation, supporting a potential durability of response.

ANCA remained detectable in approximately one‐third of patients in our cohort at week 52. This proportion appears lower than that reported in another real‐world study by Gabilan et al, in which more than half of the patients remained ANCA positive at 12 months.^7^ Differences in patient characteristics, treatment strategies, and timing of avacopan initiation may partly account for this discrepancy. A progressive reduction in inflammatory activity was also observed over time in our cohort.

The observed safety profile was consistent with prior studies.^8^, ^9^ Recently, the US Food and Drug Administration issued a Drug Safety Communication warning of cases of serious drug‐induced liver injury associated with avacopan, including rare cases of liver failure requiring transplantation.^10^ In our prospective cohort, liver safety signals were limited. Only two patients developed mild (grade 1) elevations of liver enzymes, one of whom required temporary dose reduction, and the other continued treatment without intervention. No patient experienced severe drug‐induced liver injury, acute liver failure, or treatment discontinuation due to hepatotoxicity. Although our sample size is limited and cannot exclude rare events, these findings differ from the severe hepatic events described in postmarketing pharmacovigilance and highlight the importance of regular liver function monitoring during avacopan therapy.

Overall, in our prospective real‐world cohort, AEs were generally infrequent and manageable, and treatment discontinuation due to safety issues occurred in a limited proportion of patients (15%; Table 1). Unlike Zonozi et al, the most common AEs in our cohort were infectious events, accounting for 40% of all AEs. Two deaths occurred during follow‐up in elderly patients (>70 years of age) with a long history of relapsing AAV (disease duration of 10 years). Both deaths were due to infections, underscoring the importance of close monitoring, particularly in older and immunocompromised individuals. Early access to avacopan and GC‐sparing strategies in the early phase of the disease may help mitigate this risk. Importantly, improvements in AAV‐PRO_ita questionnaire scores were accompanied by stable VDI scores throughout follow‐up, suggesting that the benefits associated with C5a receptor inhibition extended beyond disease activity control to include preservation of accumulated organ damage.

Strengths of our study include its prospective design, the inclusion of a heterogeneous real‐world population managed across expert nephrology and rheumatology centers, systematic patient monitoring, and the integration of validated patient‐reported outcome measures. Notably, to our knowledge, this is the first study to apply the Italian version of the AAV‐PRO questionnaire in patients receiving avacopan in routine clinical practice.

Nevertheless, several limitations should be acknowledged. The relatively small sample size, the absence of a comparator group, and the heterogeneity of treatment strategies across participating centers preclude causal inferences regarding the effectiveness of avacopan. In addition, treatment decisions—including GC tapering and maintenance immunosuppression—were left to physician discretion, reflecting routine clinical practice rather than a standardized protocol.

During the revision of this manuscript, the original publication of the ADVOCATE trial was retracted following concerns regarding the integrity of the underlying trial data. In addition, regulatory authorities have raised concerns regarding both the reliability of the pivotal trial data and postmarketing reports of serious liver injury associated with avacopan. Although these developments should be considered when interpreting the overall evidence base, they do not affect the integrity of the prospectively collected observational data presented in this study. Accordingly, our findings should be interpreted as independent real‐world evidence describing the clinical outcomes associated with C5a receptor inhibition in routine clinical practice.

Notably, the therapeutic relevance of complement inhibition in AAV extends beyond avacopan itself. Alternative pathway inhibitors, including the factor B inhibitor iptacopan (NCT06388941), are currently under clinical investigation. In this evolving therapeutic landscape, independent prospective real‐world studies may help contextualize emerging evidence and inform the future development of complement‐targeted strategies in AAV.

This prospective, multicenter, real‐world study showed that patients treated with avacopan achieved high remission rates, substantial GC reduction, improvement in kidney function, and meaningful QoL benefits during follow‐up. Overall, avacopan was generally well tolerated in this cohort. These findings provide independent, prospective real‐world evidence on the clinical outcomes associated with avacopan treatment in routine practice. Further independent comparative studies will be important to better define the role of complement‐targeted therapies in the management of AAV, particularly in light of the evolving therapeutic and regulatory landscape.

## AUTHOR CONTRIBUTIONS

All authors contributed to at least one of the following manuscript preparation roles: conceptualization AND/OR methodology, software, investigation, formal analysis, data curation, visualization, and validation AND drafting or reviewing/editing the final draft. As corresponding author, Dr Quartuccio confirms that all authors have provided the final approval of the version to be published and takes responsibility for the affirmations regarding article submission (eg, not under consideration by another journal), the integrity of the data presented, and the statements regarding compliance with institutional review board/Declaration of Helsinki requirements.

## Supporting information


**Disclosure Form**:
